# [RETRACTION]: Expression of serum microRNA-378 and its clinical significance in renal cell carcinoma

**DOI:** 10.1590/1678-4685-gmb-2016-0121ret

**Published:** 2020-04-06

On request received from the corresponding author, the following article is retracted because the authors noted problems in the statistical analysis of the data, so the conclusions drawn in this study cannot be sustained:

Shi, Lixin, Zhang, Lei, Wang, Chunyang, Sun, Shengkun, Cao, Xiyuan, & Zhang, Xu. (2017). Expression of serum microRNA-378 and its clinical significance in renal cell carcinoma. Genetics and Molecular Biology, 40(2), 525-529.

10.1590/1678-4685-gmb-2016-0121

Prof. Dr. Carlos Frederico Martins Menck

Editor in chief

